# A Severe Case of Tuberculosis Radiologically and Endoscopically Mimicking Colorectal Cancer with Peritoneal Carcinomatosis

**DOI:** 10.1155/2017/6206951

**Published:** 2017-10-03

**Authors:** Timo Rath, Raja Atreya, Walter Geißdörfer, Roland Lang, Andreas Nägel, Markus F. Neurath

**Affiliations:** ^1^Department of Medicine 1, Friedrich-Alexander University Erlangen-Nuremberg, Division of Gastroenterology, Endocrinology and Pneumology, Ludwig Demling Endoscopy Center of Excellence, Ulmenweg 18, 91054 Erlangen, Germany; ^2^Institute of Clinical Microbiology, Immunology and Hygiene, University Hospital of Erlangen, Friedrich-Alexander University Erlangen-Nuremberg, Wasserturmstrasse 3-5, 91054 Erlangen, Germany

## Abstract

Although generally rising in incidence, intestinal tuberculosis is still rare in western countries and due to unspecific manifestations mainly as ulcerations on endoscopy, diagnosis of intestinal tuberculosis is challenging. Within this report, we describe a case of severe intestinal tuberculosis radiologically and endoscopically masquerading as colorectal cancer with peritoneal carcinomatosis. Our case exemplifies that intestinal tuberculosis needs to be considered as a differential diagnosis in patients at risk and that undelayed and sensitive diagnosis of intestinal tuberculosis is of central importance for avoiding unfavorable disease outcome.

## 1. Introduction

Tuberculosis (TB) represents a major health problem in developing countries and while also many developed countries face a resurgence of TB, intestinal tuberculosis is still rare in western countries [1, 2]. Due to unspecific manifestations mainly as ulcerations on endoscopy [1–3], diagnosis of intestinal tuberculosis is challenging, especially when pulmonary infection is absent [4, 5]. Further, intestinal tuberculosis can mimic various abdominal pathologies including Crohn's disease, periappendiceal abscess, ischemic colitis, tumors, or intestinal infections other than TB [6, 7]. Although colonoscopy with biopsies is the procedure of choice for the diagnosis of intestinal TB, even histopathologic diagnosis can be difficult especially when granulomas are absent.

Herein, we report about intestinal tuberculosis radiologically and endoscopically masquerading as colorectal cancer with peritoneal carcinomatosis. Our case exemplifies that a high level of suspicion for intestinal tuberculosis is critical in patients at risk and that rapid and sensitive diagnosis is important to avoid unfavorable disease outcome.

## 2. Case Report

**Figure 1:**
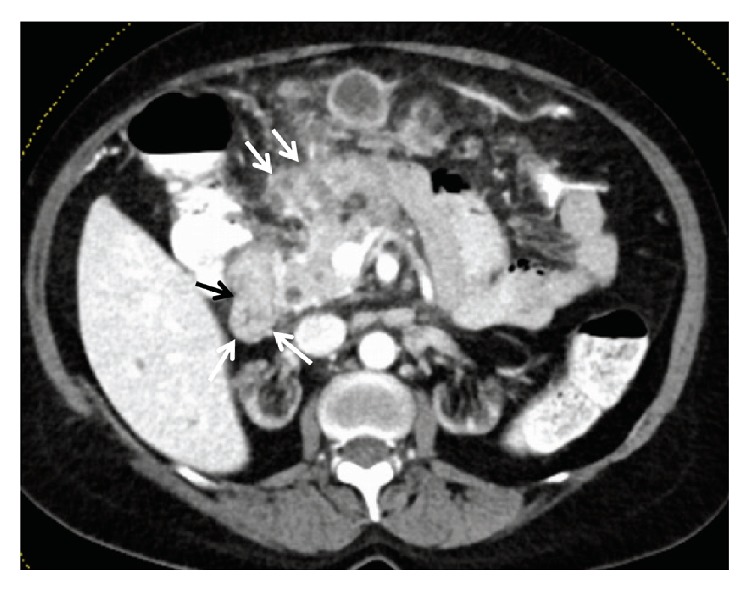
CT scan with multiple contrast-enhancing peritoneal nodules and large intraperitoneal soft-tissue masses (arrows), radiologically impressing as severe peritoneal carcinomatosis.

**Figure 2:**
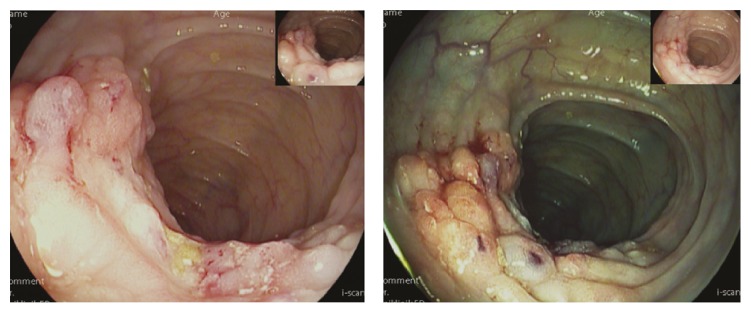
Polypoid-tumorous lesion with irregular mucosa, superficial hemorrhages, contact bleeding, and a central ulceration at the hepatic flexure on virtual chromoendoscopy with i-scan.

**Figure 3:**
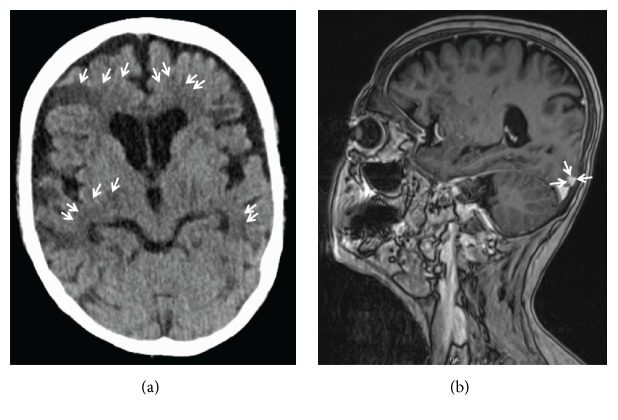
Bilateral ischemic lesions (CT, (a) arrows) and contrast-enhancing lesions directly adjacent to the dura mater (MRI, (b) arrows), radiologically consistent with multiple septic emboli and tuberculosis manifestation within the CNS.

A 51-year-old kidney transplanted patient was admitted to the department of nephrology with abdominal pain and significant weight loss for several weeks. CT scan showed multiple contrast-enhancing peritoneal nodules and large intraperitoneal soft-tissue masses radiologically impressing as severe peritoneal carcinomatosis (Figure 1), a hypodense lesion within the right adrenal gland radiologically appearing as a metastasis as well as enlarged retroperitoneal lymph nodes, while the lung was unremarkable on CT scan. The patient was then referred to our endoscopy unit for tumor screening. Colonoscopy with virtual chromoendoscopy (i-scan) revealed a polypoid lesion at the hepatic flexure with irregular mucosa, hemorrhages, contact bleeding, and a central ulceration (Figure 2). On histopathology, no dysplasia or cancer was found. Importantly, histopathology was also negative for the presence of granulomas. A second colonoscopy for targeted biopsies was initiated and histopathology was again negative for cancer; however, subsequently performed Ziehl-Neelsen stain revealed acid-fast bacteria. In a third colonoscopy, biopsies for microbiological analysis and resistance testing were obtained and DNA of rifampicin-sensitive* M. tuberculosis* complex was detected using the Xpert MTB/RIF assay on the GeneXpert Dx System (Cepheid, Sunnyvale, CA) on the same day. Immediately thereafter, first-line antituberculostatic treatment was initiated. Liquid culture (BACTEC™ MGIT™, Becton Dickinson, Franklin Lakes, NJ) was positive after 4 days. The strain was identified as* M. tuberculosis* based on* gyrB* sequence analysis [8] and phenotypically susceptible to all first-line tuberculostatic drugs. The subsequent course of disease was complicated by episodes of epileptic seizures with aphasia and hemiparesis. Cranial CT and MRI showed bilateral ischemic lesions and contrast-enhancing lesions directly adjacent to the dura, radiologically consistent with multiple septic emboli and tuberculosis manifestation within the CNS (Figure 3). Under tuberculostatic therapy, neurological symptoms slowly ameliorated; however, organic brain syndrome persisted.

## 3. Discussion

The incidence of abdominal TB has been steadily increasing in the past 20 years [6, 9, 10]. At the same time, diagnosis of intestinal tuberculosis remains challenging, especially when active pulmonary infection is absent. The current hurdles in the diagnosis of intestinal TB are mainly based on its unspecific clinical and endoscopic presentation and histopathology that frequently misses the pathognomonic lesions. As a result, time to diagnosis can range from 2 days to 11 months with a median time to diagnosis of 50 days, as indicated by data from a North American hospital [11]. In light of this, diagnosing intestinal tuberculosis requires a high index of suspicion especially in countries in which tuberculosis is not endemic and an increased awareness of intestinal tuberculosis as a severe differential diagnosis seems critical especially in patients at risk in order to warrant undelayed treatment initiation.

Intestinal TB is predominantly located in the ileocecal region and the presence of lymphoid tissue within the ileum and a physiologic stasis facilitating prolonged contact between bacteria and the mucosa have been discussed as reasons for the ileum and cecum being the most common sites of disease manifestation [1–3, 6].

Endoscopically, intestinal tuberculosis typically appears as ulcerations, nodules, or luminal narrowing [1–3] and the clinical presentation is usually nonspecific with fever, weight loss, abdominal pain, and changes of bowel habits as the most commonly observed symptoms [2, 7].

To date, endoscopy with biopsies is considered the procedure of choice for the diagnosis of intestinal TB. In addition to the diagnostic dilemma of rather unspecific appearance on endoscopy and eventually vague clinical symptoms as outlined above, the pathognomonic features of epithelioid granulomas with Langhans giant cells, central caseous necrosis, and presence of acid-fast bacilli are identified only in a minority of biopsy specimens [12, 13]. In fact, granulomas with or without caseation are present in less than 50% of patients [7, 14, 15], while clusters of epithelioid cells without well-formed granulomas are only observed in 20–30% of the biopsies [14, 15]. In contrast, a large percentage of patients with colonic tuberculosis histology reveals only chronic nonspecific changes in the form of chronic inflammatory cells in the lamina propria [2, 13]. This diagnostic dilemma is very well reflected in our patient, in which the biopsies obtained during the first colonoscopy did not exhibit pathognomonic features of intestinal TB. Therefore, a second colonoscopy was necessary in which the presence of acid-fast bacteria confirmed mycobacterial infection.

Culture of biopsy material remains the gold standard for the diagnosis of intestinal tuberculosis but usually requires 4 to 6 weeks until results are obtained [6, 7]. Furthermore, studies in patients with colonic tuberculosis indicate that positive culture can be found in only one-third of patients or even less [15, 16].

In contrast, PCR for* M. tuberculosis* from biopsies provides rapid diagnosis of TB and positive predictive values up to 100% have been reported [17]. Although more sensitive than culture and acid-fast stains in diagnosing intestinal TB [18], recent data reported lower negative prediction and variable specificity in extrapulmonary TB [19, 20]. Nevertheless, the possibility of obtaining results within 48 hours by itself confers a major advantage over histopathology and thus should be considered in patients at risk for intestinal TB such as HIV-infected individuals and immunosuppressed or immunocompromised patients.

In summary, this case exemplifies the fact that intestinal tuberculosis can radiologically and endoscopically mimic colorectal cancer. Further, as illustrated within this case, a high level of suspicion is necessary in patients at risk in which PCR analysis of a colonic biopsy might be considered initially and in parallel to conventional histopathology for sensitive and fast differential diagnosis and undelayed treatment initiation in order to avoid unfavorable disease outcome.
